# Arterio-Venous Aneurysm

**Published:** 1902-05-17

**Authors:** 


### ARTERIO-VENOUS ANEURYSM.

In times gone by arterio-venous aneurysm was almost always the result of punctured wounds—an accidental puncture of the brachial artery during the process of bleeding from the median basilic vein or a stab with a knife or dagger. With the less frequent use of knives in brawls, and of blood-letting in therapeutics, such punctured wounds have become less common, and thus arterio-venous aneurysms have become very rare. Now, however, says Sir Frederick Treves,[1] the introduction of bullets of small calibre into warfare has made these aneurysms as familiar as ever, and in the surgical records of the war in South Africa numerous examples of this condition are to be found. The only operative measure which is to be recommended in these cases, he says, is the ligature of the vessel or vessels at the wounded spot. The ideal procedure is that in which both the affected artery and the vein are ligatured above and below the point of communication. But this it is obvious cannot always be done. When the condition is that of varicose aneurysm it is as well that the sac between the vessels should also be excised. In the four cases described by Sir Frederick Treves the injury was in each case inflicted by a bullet, by a Mauser in three cases, and by a Lee-Metford in the fourth, and in all four examples the bullet penetrated the part, escaping without damaging any bone, and the resulting wound healed by first intention. It was quite impossible to diagnose the precise character of the arterio-venous communication before the part was exposed by operation, and it is, he thinks, doubtful whether the precise differential signs which are detailed as means for distinguishing one form of arterio-venous aneurysm from another are met with with any frequency in actual practice. In the first two cases the condition found was that of varicose aneurysm (in one involving the superficial femoral vessels and in the other the popliteal artery at its point of bifurcation), and both were cured by ligature of the vessels, the sac being also excised in one case. In the third and fourth cases an aneurysmal varix was discovered, and in neither of these was it found possible to carry out the perfect operation by ligature of the involved vessels both above and below the point at which they had been wounded. Yet—and this is the important point—notwithstanding the fact that in each of these cases the results of the operation came short of complete perfection, in both they were extremely satisfactory in comparison with the condition for the relief of which the operations were performed. In the one case the point of communication between the artery and vein, or veins, was precisely at the bifurcation of the common femoral. Having tied the common femoral above the communication, and the superficial femoral below, it was obviously out of the question to apply a ligature to the profunda artery, nor, having regard to the future of the limb, was it thought well to ligature any of the veins which were exposed. The operation therefore consisted solely in the ligaturing of the common femoral and the superficial femoral arteries. Yet the patient did well, and although there was some slight return of pulsation and the bruit and thrill remained, there was no swelling or œdema and no discomfort, the patient becoming able to ride and walk well, the only thing complained of being that after long or unduly quick walking he experienced a pain in the calf. In the last case two operations were performed, in the second of which it seemed that the communication concerned the internal maxillary artery at some distance behind the ramus of the jaw, and was inaccessible without dividing that bone—a proceeding which was not considered desirable under the circumstances, the whole area being obscured with dilated and pulsating veins and the risks from hæmorrhage being very great. The external carotid was therefore ligatured on the proximal side of the origin of the internal maxillary, and ligatures were also placed upon the temporal arteries and on the veins issuing from behind the mandible. In the previous operation the external carotid had been divided just beyond the origin of the superior thyroid, which artery, together with the facial, had been tied. Thus the blood supply was cut off from the affected area as far as possible. In this case also, although the cure was not absolute, the improvement was immense; in fact, the patient described himself as "very well indeed." These cases serve well to show how important a field there is for surgery in cases of arterio-venous aneurysm. In some, as in the first two here mentioned, the whole thing may be removed and a perfect cure may result; but even in those in which the operation of perfection may prove impossible, an immense amelioration of the patients' conditions may be produced by modifying the circulation through the parts.

[1] Brit. Med. Jour., May 10.

